# Cardiac tamponade: an uncommon presenting feature of systemic lupus erythematosus (a case-based review)

**DOI:** 10.11604/pamj.2020.36.368.25044

**Published:** 2020-08-28

**Authors:** Chadia Chourabi, Houaida Mahfoudhi, Sameh Sayhi, Rim Dhahri, Karima Taamallah, Sarra Chenik, Abdeddayem Haggui, Nadhem Hajlaoui, Dhaker Lahidheb, Ajili Faida, Nadia Ben Abdelhafidh, Bassem Louzir, Wafa Fehri

**Affiliations:** 1 Department of Cardiology, Military Hospital of Tunis, Tunis, Tunisia,; 2 Department of Internal Medicine, Military Hospital of Tunis, Tunis, Tunisia

**Keywords:** Systemic lupus erythematosus, tamponade, pericardial effusion

## Abstract

Although pericarditis is the most prevalent cardiac involvement in systemic lupus erythematosus (SLE), cardiac tamponade is extremely infrequent notably as the first manifestation of the disease. Here we report the case of a 22-year-old woman presenting with cardiac tamponade as the initial presentation of SLE.

## Introduction

Systemic lupus erythematosus (SLE) is a chronic autoimmune disease that has many clinical manifestations and may involve any organ system. Cardiac involvement is frequent and can be observed in more than 50% of patients with SLE [1]. Although pericardial effusions and pericarditis are the most prevalent cardiac manifestation, cardiac tamponade is extremely rare, especially as the first manifestation of the disease [1]. Here we report a case of cardiac tamponade as the initial presentation of SLE.

## Patient and observation

**Figure 1:**
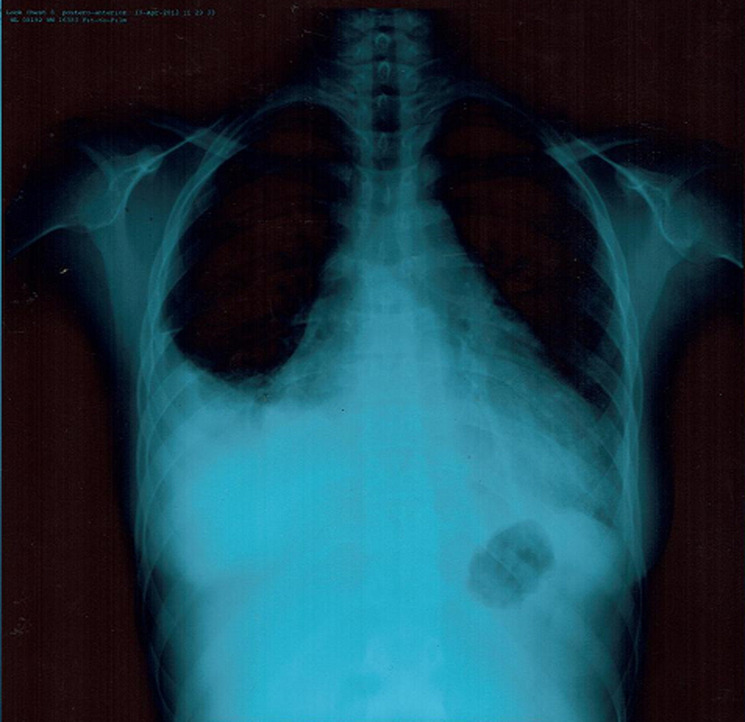
anteroposterior chest radiography showing enlargement of the cardiac silhouette with a right-sided pulmonary effusion

**Figure 2:**
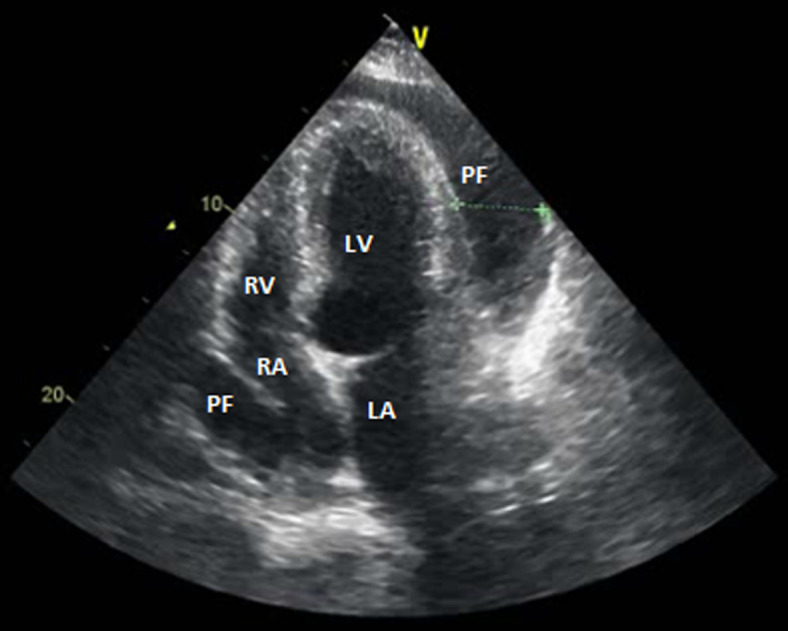
echocardiography apical 4 chamber view showing large pericardial effusion and collapse of the right atrium (RA), RV: right ventricle, LV: left ventricle and PF: pericardial fluid

A 22-year-old woman, without any formerly diagnosed diseases, presented to the emergency department with a 3 day history of dyspnea, fatigue and central chest pain. She reported increasing dyspnea that developed in the previous month. She also had intermittent pain in the small joints for several months. There was no family history of SLE. On examination, she had a temperature of 38.2°C, a heart rate of 115 beats/minute, blood pressure of 90/60mmHg, respiratory rate of 25 breaths/minute, muffled heart sounds and jugular distention. The electrocardiogram showed sinus tachycardia and low voltage. The chest radiograph showed an enlargement of the cardiac silhouette with a right-sided pulmonary effusion (Figure 1). The echocardiography revealed a large circumferential pericardial effusion (Figure 2), with diastolic collapse of the right atrium, dilated inferior vena cava and 30% respiratory variation of the Doppler mitral valve, confirming the diagnosis of cardiac tamponade. Initial workup showed normochromic normocytic anemia with hemoglobin at 8.5 g/dl, CRP: 75 mg/L, renal and liver function tests were normal.

Emergency pericardiocentesis was indicated but could not be performed because of the incapability to place the guidewire into the pericardial cavity. So, urgent surgical pericardiectomy with window procedure was realized, 1200 of clear fluid was evacuated. The pericardial fluid contained 2900 white blood cells/mm^3^, 100 red blood cells/mm^3^, bacteriological culture and cytologic examinations yielded negative results. The pericardial biopsy showed features of nonspecific inflammation, malignancy and tuberculosis were ruled out. Further investigations were accomplished to determine the etiology. Immunological workup revealed antinuclear antibodies titer of 1: 1280, positive anti-SSA and anti-SM antibodies and hypocomplementemia. The coombs test was positive. The diagnosis of systemic lupus erythematosus was established based on hemolytic anemia, serositis, arthralgia, positive anti nuclear and anti-SM antibodies and low complement. The patient was started on intravenous therapy with methylprednisolone followed by prednisone 60mg by mouth daily with hydroxychloroquine. She had a good clinical response and control echocardiography showed complete resolution of the pericardial effusion without recurrence.

## Discussion

Our patient had cardiac tamponade as the initial presentation of SLE. SLE is an autoimmune disease that can affect any organ system. Heart involvement is commonly observed. Pericardial effusion is one of the most prevalent manifestations of SLE found in about 50% of patients and generally, it tends to be small and hemodynamically insignificant [2]. Nonetheless, tamponade is rare, estimated to occur in fewer than 1% of patients with SLE [3]. Cardiac tamponade as the initial disease presentation of the disease is even rarer. To our knowledge, only isolated cases and small series on the subject have been related. Drug-induced lupus syndrome with hydralazine, procainamide, isoniazid and carbamazepine was reported to present initially as tamponade [4-8]. In patients with known SLE, cardiac tamponade was more described in women and in patients with anemia, renal disease, pleuritis, higher ESR values and lower C4 levels [1, 2]. More recently, in a series of 409 patients with SLE of whom 24 developed tamponade, Goswami *et al*. reported that pleuritis, anti-nucleosome antibodies and the size of pericardial effusion were found to be significant predictors of tamponade [9]. Among these factors, our patient was female, had anemia, large pericardial effusion, pleuritis and low C4 level. In the vast majority of cases, treatment consists of high doses of glucocorticoids and hydroxychloroquine after urgent pericardial fluid withdrawal. Generally, a favorable evolution after treatment was noted, recurrent effusions and pericardial thickening were reported in two patients in the series of Kahl *et al*. [2]. The evolution to constriction is extremely rare [10].

## Conclusion

Cardiac tamponade is a life-threatening condition, SLE should be considered as a possible etiology. This case is reported for the rareness of cardiac tamponade as the first manifestation of SLE and for the fact that a pericardial window was realized, which is extremely rare in SLE.
